# Hydrogen Sulfide Prevents Mesenteric Adipose Tissue Damage, Endothelial Dysfunction, and Redox Imbalance From High Fructose Diet-Induced Injury in Aged Rats

**DOI:** 10.3389/fphar.2021.693100

**Published:** 2021-08-30

**Authors:** Oleh Revenko, Yaroslav Pavlovskiy, Maryana Savytska, Antonina Yashchenko, Vasyl Kovalyshyn, Ilona Chelpanova, Olena Varyvoda, Oksana Zayachkivska

**Affiliations:** ^1^ Department of Physiology, Danylo Halytsky Lviv National Medical University, Lviv, Ukraine; ^2^ Department of Histology, Cytology and Embryology, Danylo Halytsky Lviv National Medical University, Lviv, Ukraine; ^3^ Department of Pathological Anatomy and Forensic Medicine, Danylo Halytsky Lviv National Medical University, Lviv, Ukraine

**Keywords:** white adipocyte, mitochondria, mesentery, H_2_S (hydrogen sulfide), high fructose consumption, age-related, NaHS

## Abstract

A high fructose diet (HFD) and advanced age are key factors for the gradual loss of physiological integrity of adipose tissue. Endogenous hydrogen sulfide (H_2_S) has beneficial effects on cytoprotection and redox balance. But its interactive effects on age-related damage of mesenteric vessels and connective and adipose tissues (MA) during HFD which could be the base of the development of effective physiological-based therapeutic strategy are unknown. The aim of study was to investigate age- and HFD-induced mesenteric cellular changes and activities of enzymes in H_2_S synthesis and to test the effects of sodium hydrosulfide (NaHS) which is considered an H_2_S donor on them. Adult and aged male rats on a standard diet (SD) or 4-week HFD were exposed to acute water-immersion restraint stress (WIRS) for evaluation of mesenteric subcellular and cellular adaptive responses by electron microscopy. The effects of exogenous NaHS (5.6 mg/kg/day for 9 days) versus vehicle on mesentery changes were investigated. Serum glucose level, thiobarbituric acid reactive substances (TBARS), and activities of cystathionine γ-lyase (CSE) and cystathionine β-synthase (CBS), thiosulfate-dithiol sulfurtransferase (TST), and sulfite oxidase (SO) were examined by spectrophotometry. In both adult and aged SD groups, treatment with NaHS protected mesenteric cells after WIRS. In both groups, the treatment with NaHS also protected MA mitochondria, microvascular endothelial and sub-endothelial structures, and fibroblasts versus the vehicle-treated group that had signs of damage. HFD increased MA injury and mitochondrial changes in both aged and adult rats. HFD-associated malfunction is characterized by low activities of CSE, CBS, TST, SO, and increased TBARS. Finally, we demonstrated that pretreatment with NaHS inhibited MA and mitochondria alterations in aged rats exposed to HFD and WIRS, lowered TBARS, and enhanced H_2_S enzyme activities in contrast to the vehicle-treated group. Mitochondrial integrity alterations, endothelial damage, and redox imbalance are key factors for rat mesenteric adipose tissue damage during advanced age. These alterations and MA hypertrophic changes retain the central for HFD-induced damage. Moreover, H_2_S signaling contributes to MA and mitochondria redox balance that is crucial for advanced age and HFD injury. The future study of H_2_S donors’ effects on mesenteric cells is fundamental to define novel therapeutic strategies against metabolic changes.

## Introduction

There is growing evidence that suggests the importance of the functional roles of adipose tissue. This includes white adipocytes, brown adipocytes, and beige adipocytes, which differ in morphology and functions (Hilton et al., 2015; Shimizu et al., 2015; Lee et al., 2019). These cells are unique in their ability to collect and integrate thousands of different types of input and to translate them into signaling pathways that are responsible for pleiotropic expression contributing to the risk of numerous metabolic disorders related to obesity, type 2 diabetes, and gastrointestinal and cardiovascular diseases processes (Li et al., 2018; Scheja et al., 2019; Zhu et al., 2019). Recent clinical data has shown that markers of both obesity and the COVID-19 had severe negative outcomes when these cofactors are present in older age, leading to multi-organ dysfunction (Korakas et al., 2020; Tamara et al., 2020).

Since the visceral, gonadal, and subcutaneous white adipose tissues have different physiological and metabolic functions and adaptive potential, these tissues have diverse roles in metabolism regulation (Wronska and Kmiec, 2012; Do et al., 2019). It is widely understood that maintaining mitochondrial homeostasis and its quality is crucial for varied signaling pathways for cytoprotection, apoptosis, or inflammation (Cedikova et al., 2016; Kiriyama et al., 2018; He et al., 2020). Moreover, ultrastructural studies of white adipocytes are helpful to determine the shape, quality, and quantity of their mitochondria, focusing on them as a target for novel therapy strategies (Miliotis et al., 2019). The recent discovery has noted visceral fat tissue that contains white adipocytes physiologically active in the mesentery, newly described as separate organs in the abdominal cavity (Coffey et al., 2020). Adipocytes in the mesentery (MA) have a poor blood capillary supply and intrinsically low antioxidant enzyme defenses which make them vulnerable to hypoxia and free radical damage (Kredel et al., 2014). Since it has been reported that the accumulation of a mesenteric adipocyte tissue (known as creeping fat) is the driving force for transmural inflammation, interaction MA and fibrosis may play role in the pathogenesis of several diseases, like Crohn’s disease (Mao et al., 2019; Rivera et al., 2019). However, the exact mechanism of adipose tissue damage which is defined as the gradual loss of physiological integrity with both quantitative and qualitative changes in adipocyte numbers and stromal-vascular cell composition is not yet entirely understood (Wang et al., 2018; Conte et al., 2019; Streich et al., 2020). Recently, in the previous study, we have obtained results of MA changes during exposure to a high fructose diet (HFD) (Revenko et al., 2020). Moreover, the link between mesenteric white adipocytes damage during aging and the chronic overload nutrition of glycemic carbohydrates still remains incomplete; thus, in this report, we demonstrate that mesenteric cells can change in advanced age and have compared their change during HFD.

The vasodilatory, anti-inflammatory, antioxidant properties of endogenous hydrogen sulfide (H_2_S) have beneficial effects as H_2_S donors on cytoprotection during metabolic dysregulation in numerous studies (Suzuki et al., 2011; Shibuya and Kimura, 2013). The impact of H_2_S signaling and redox balance on mitochondria has recently been demonstrated as a critical control point between the physiological and pathological states (Murphy et al., 2019; Paul et al., 2020). However, little is known about the effect of H_2_S on mitochondria dynamics in mesenteric white adipocytes during metabolic states related to aging and the chronic overnutrition of glycemic carbohydrates which has a crucial impact on oxidative damage (Mezouari et al., 2020). Recently, it has been shown that chronic fructose overload causes metabolic disorders and comorbidities (Bidwell, 2017; de Farias Lelis et al., 2020). There is a pressing need for translational research to study the link of MA damage as the gradual loss of physiological integrity and redox system during aging and the chronic overnutrition of glycemic carbohydrates. This will help develop effective target-focused therapy. Since H_2_S may also control the processes important for redox balance, we hypothesized that, by studying biomarkers of lipid peroxidation products based on levels of thiobarbituric acid reactive substances (TBARS) and activities of enzymes involved in H_2_S synthesis, we will clarify the mechanism of H_2_S effects on mesenteric white cells during aging and in an experimental metabolic model using rat fed with high fructose diet (HFD). These enzymes include cystathionine γ-lyase (CSE), cystathionine β-synthase (CBS), thiosulfate-dithiol sulfurtransferase (TST), and sulfite oxidase (SO) which control the redox system on an intracellular and intercellular basis. Thus, the objectives of this study are 1) to have a closer look at early age-related changes in mesenteric adipocytes in rats fed with SD and during HFD and 2) to investigate mesenteric white adipocyte tissue damage characteristics focusing on the mitochondria-centered picture during stress induction and stimulation of the endogenous H_2_S bioavailability by exogenous NaHS administration.

## Methods

### Ethical Approval

All experiments were approved by the local animal care committee at the Danylo Halytsky Lviv National Medical University Ethics Committee (protocol April 23, 2018, № 4) and were carried out in accordance with the National Institute of Health Guide for the Care and Use of Laboratory Animals (NIH Publications No. 80-23) revised 1996 or the United Kingdom Animals (Scientific Procedures) Act 1986 and associated guidelines, or the European Communities Council Directive of November 24, 1986 (86/609/EEC). All efforts were made to minimize animal suffering and to reduce the number of animals used.

### Overall Study Design and Experimental Animals

**FIGURE 1:**
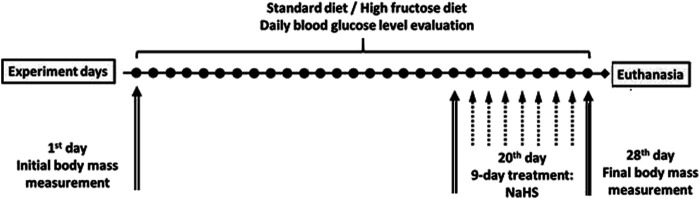
Overview of the study.

All experiments were performed on male adult (age = 12–14 weeks, *n* = 36) and aged Wistar rats (age = 42–46 weeks, *n* = 36). Animals were maintained under a constant 12 h light and dark cycle and an ambient temperature of 21–23°C with 50 ± 10% relative humidity. All animals were kept in raised mesh-bottom cages to prevent coprophagy and subdivided into groups (*n* = 6). Figure 1 shows the design of the study. Rats were randomly assigned to nine experimental groups. Adult and aged rats in the control groups had free access to water and were fed a standard diet (SD). Animals in the experimental groups were fed a high fructose diet (HFD) by receiving 28-day unrestricted access to a 40% solution of fructose ad libitum and fed at the same time SD (Pavlovskiy et al., 2020). To investigate adaptive reactions to acute injury on the 29th day of study, acute stress was induced by the model of Takagi Takagi and Okabe (1968) that involves short-term exposure to water-immersion restraint stress (WIRS). For this intervention, the rats were placed in restraint cages and immersed vertically to the level of the xiphoid process in a water bath of 23°C for 3.5 h. Food deprivation for 12 h before the end of experiments has been performed for all rats. Daily animal health checks were performed by laboratory or institutional laboratory animal staff, under the supervision of the institutional veterinarian.

Weights were recorded at the beginning and end of the study by an RN 10C13U, 100 g–10 kg, ±5 g (Vaga, Kyiv, Ukraine). Rat blood glucose concentrations were measured daily after 15 h of fasting (18:00–9:00) by a glucometer (Achtung TD-4207, Munich, Germany) using a blood sample from the tail vein. The data in each group were compared at the beginning and end of the study and with the results from control rats. At the end of the experiment, the rats were deeply anesthetized with an intramuscular injection of ketamine (60 mg/kg; Biovet, Bila Tserkva, Ukraine) and sacrificed, and after that, blood was collected and the samples of the mesentery tissue associated were resected. After thoroughly washing with saline, sections of the mesentery were taken for histological examination and the establishment of macroscopic signs of damage. Samples for histological cellular and subcellular analysis by electron microscopy were obtained from the mesentery associated with the small intestine.

#### The Cellular and Subcellular Investigation *via* Electron Microscopy

We directed electron microscope evaluation of the mesentery to assess adipocytes, vascular changes, fibroblasts, and collagen fibers. For the cellular and subcellular analysis, the mesenteric material was fixed with a 2% solution of osmium oxide (OsO_4_) eV 0.10 mol/L phosphate buffer. Subsequently, mesenteric material was processed according to generally accepted methods.

Ultrathin sections (30–60 nm) were made using an ultramicrotome UTMTP-3M (Sumy Electron Optics PKF, Sumy, Ukraine). After Reynolds staining, sections were photographed and examined using an electron microscope «UEMV-100K» (Sumy Electron Optics PKF, Sumy, Ukraine) at a magnification of 4,000, 6,000, and 10,000x. Histological analyses were performed by at least two independent people blinded to the identity of the samples. About 15 different cells in each sample were analyzed per rat. A single researcher that was unaware of the experimental groups performed the analysis. The state of histopathological mesentery changes in each group in comparison with that of the other groups was determined by protocol-blinded researchers.

#### Biochemical Analysis

Blood glucose concentrations were measured daily by a glucometer (Achtung TD-4207, Munich, Germany) using a blood sample from the tail vein.

On the 29th day of the study, animals were sacrificed, and the samples from rat blood and mesentery which belong to the intestinal mucosa were evaluated for the serum TBARS levels and catalytic activities of CBS (EC 4.2.1.22), CSE (EC 4.4.1.1), SO (EC 1.8.3.1), and TST (EC 2.8.1.5). The resected material was washed with cold 1.15% potassium chloride solution, after which the mucous membrane was separated and homogenized in a medium of 1.15% potassium chloride in a ratio of 1:4. The mesenteric homogenates were centrifuged at 600 g and 40°C for 30 min to obtain a post-nuclear fraction.

#### Determination of Metabolic and Redox Balance Parameters by TBARS Levels and CBS, CSE, SO, and TST Activities

Plasma TBARS levels, as a biomarker of systemic effect of lipid peroxidation and oxidative damage Montes-Nieto et al. (2017), were evaluated by assaying reaction with thiobarbituric acid. The resulting lipid peroxidation products from a red-stained complex are extracted with butanol. The test tubes containing the serum were cooled at room temperature and maximum light absorbance was measured at 535 nm using a UV-visible spectrophotometer (Apel PD-303, Saitama, Japan) (Zaichko et al., 2009; Zaichko et al., 2014). We evaluated CBS, CSE, SO, and TST activities in mesenteric homogenates (nmol/min*1 mg of protein), using a modified version of the Stipanuk M.H. and Beck P.W. method as previous (Stipanuk et al., 1982; Pavlovskiy et al., 2020). Substrate and cofactor concentrations, pH, and incubation time, which could provide optimal conditions for enzyme activity determination, were selected in advance.

### Treatment Groups

The animals were subdivided into control groups of adult rats and aged rats with consuming normal rodent chow (SD) and experimental groups receiving 28-day hypercaloric HFD, without and with acute stress. To evaluate the role of H_2_S from the 19th day of the experiment, both adult and aged animals group days were treated for 9 days intragastrically by saline (as the vehicle), with NaHS at a dose of 5.6 mg/kg/day and NaHS, 5.6 mg/kg/day and stress induction. The administration of NaHS was performed in doses tested previously.

### Data and Statistical Analysis

All results were evaluated using Statistical Analysis System and visualization program « Statistica 7.0» (StatSoft, Informer Technologies, Inc.) and expressed as mean ± standard deviation for a series of experiments. A paired Mann–Whitney *U* test was used for comparisons of paired treatments between two groups, and one-way ANOVA using Dunnett’s test was performed to compare different experimental groups with control. Statistical significance was set to *p* values ≤0.05.

## Results

### Effect of 4-Week High Fructose Diet on Adult and Aged Rats Fed With Standard Diet and High Fructose Diet

Basal metabolic characteristics from adult and aged rats fed with SD were body weight: 198 ± 20 g and 256 ± 28 g and fasting glucose: 6.3 ± 0.2 nmol/L and 6.5 ± 0.2 nmol/L, respectively. Aged rats’ basal body weight was 29% more than adult rats (*p* < 0.05). There were no differences in adult rat body weight between the vehicle group and supplemented NaHS. At the end of the experiment rats fed with HFD had 32% increased final body weight in the aged group in comparison to the adult group (*p* < 0.05). Administration of NaHS did not affect final adult rat body weight fed with HFD. There were no differences in adult animals fed SD basal fasting glucose between groups supplemented with vehicle and NaHS. After 4 weeks of HFD administration in adult animals, fasting glucose levels were increased by 25% (*p* < 0.05) versus adults on SD; however, no differences were noted between the vehicle group and supplemented NaHS. At the end of the experiment, aged rats fed HFD exhibited increased fasting glucose levels by 29%. NaHS also did not affect final fasting glucose in aged rats fed with HFD.

Rats fed with HFD for 28 days exhibited an elevation of fasting blood glucose levels (from 6.3 ± 0.2 mmol/L to 7.9 ± 0.7 mmol/L for adult rats; from 6.5 ± 0.3 mmol/L to 8.4 ± 0.7 mmol/L for aged rats (*p* < 0.05 vs. rats with SD)) and about 67% (adult rats) and 71% (aged rats) gain in body weight (342 ± 31 g for adult rats; 451 ± 32 g for aged rats) over that of the control rats with SD (200 ± 21 g for adult rats; 270 ± 28 g for aged rats; *p* ≥ 0.001).

#### The Ultrastructural Differences of Mesenteric White Adipocytes and Microvessels in Adult and Aged Rats

**FIGURE 2:**
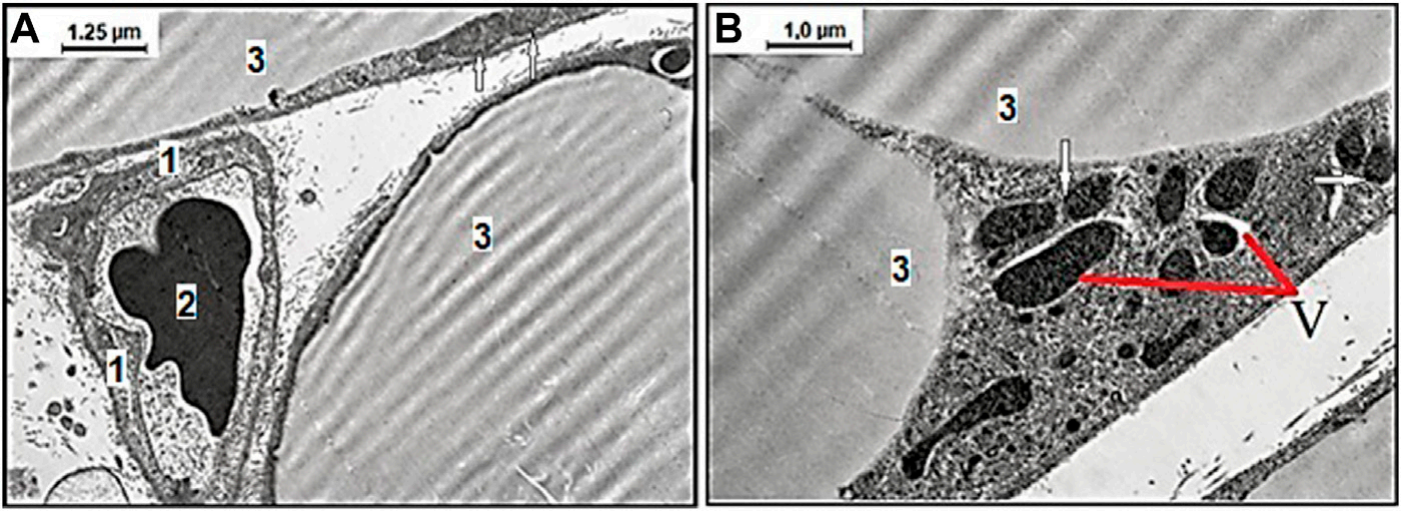
Representative electron microscopy images of the age-related effect of ultrastructural examination of mesenteric material harvested from the area associated with the small intestine of adult **(A)** and aged **(B)** rats with the standard diet. **(A)** Among the connective tissues, there are well preserved capillary endothelial cells (CECs), 1) with erythrocyte 2) in the lumen surrounded with few adipocytes 3) without signs of fat fragmentation, and normal mitochondria (arrows mark) (original magnification × 8,000). **(B)** The degenerating adipocyte with signs of vacuolization in the cytoplasm (V) and differently shaped electron-dense mitochondria. The defective mitochondria in an act of cross talk with other mitochondria (arrows mark) (original magnification × 10,000).

The obtained mesenteric material of adult and aged rats from the area associated with the small intestine fed with SD and treated with vehicle exhibited age-related differences in ultrastructural appearance represented in Figure 2. The representative photomicrographs of few adipocyte fragments without signs of fat fragmentation and well-preserved capillary endothelial cells (CECs) with erythrocyte in the lumen in adult rats fed with SD demonstrated data in Figure 2A. Examination of the mesenteric material of aged rats on SD revealed the degenerating adipocyte with signs in the cytoplasm of fat fragmentation and different shaped mitochondria demonstrated in Figure 2B. This image shows the age-related different mitochondrial morphological changes, including the round-shaped mitochondria in the act of cross talk with other mitochondria in the cytoplasm of white adipocytes.

#### Effect of H_2_S on Ultrastructural Changes of Mesenteric White Adipocytes, Microvessels, and Connective Tissue in Aged Rats Exhibited 4-Week HFD and Acute Stress

**FIGURE 3:**
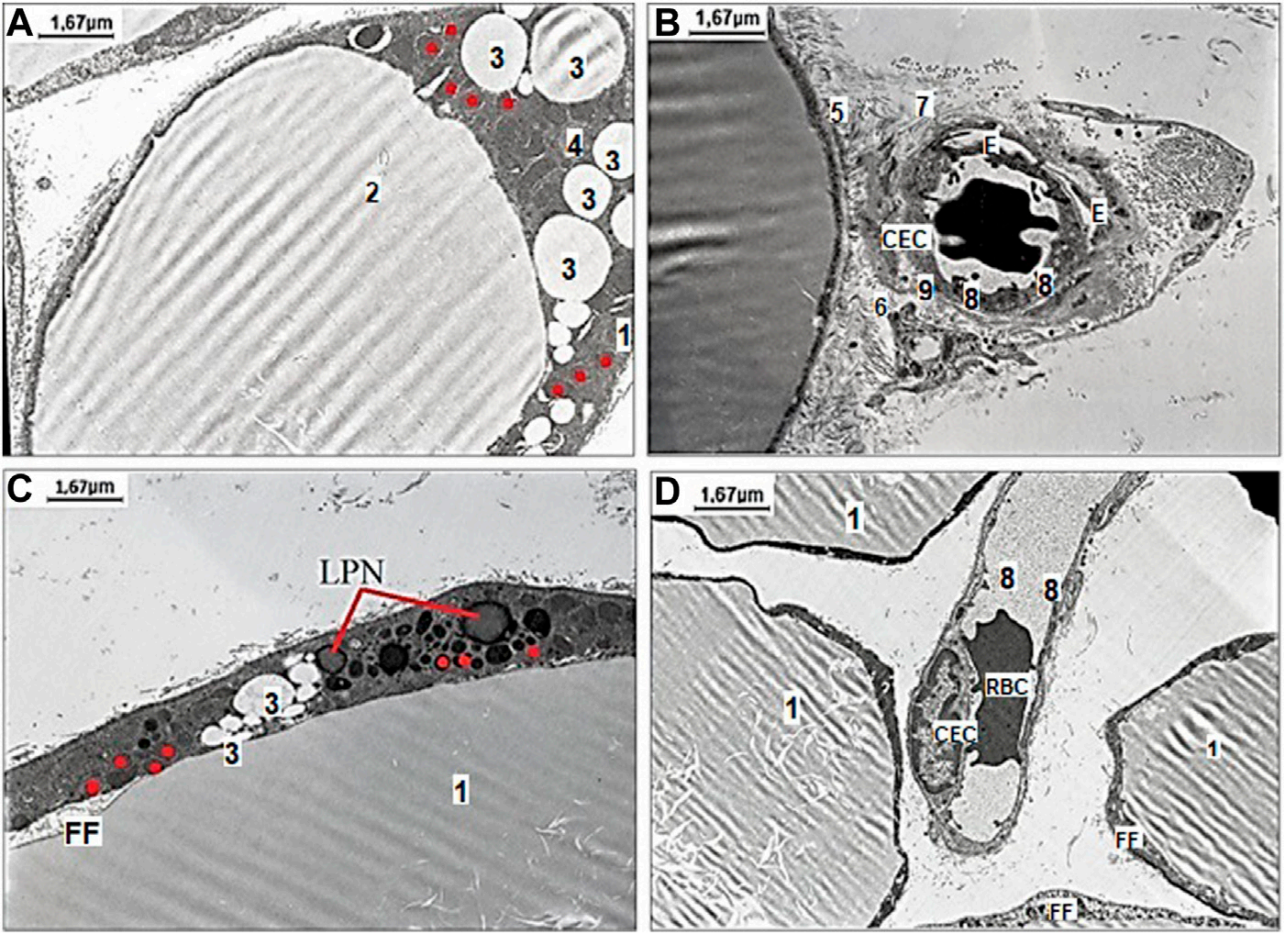
Representative pictures of the effect of fructose-feeding and stress induction on ultrastructural examination of mesenteric material harvested from the area associated with the small intestine of aged rats with vehicle **(A–B)** and NaHS **(C,D)** treatment. **(A)** The degenerating adipocyte 1) with signs of fat fragmentation 2), with small lipid drops 3) in the cytoplasm 4) of adipocytes and different shaped mitochondria (original magnification × 6,000). **(B)** Evidence of adipocyte damage includes disrupted basal membrane 5), cell debris in the interstitium 6), disorganized collagen fibers 7), and changes the microarchitecture of the capillary endothelial cells (CEC) with microvilli 8), destruction of basal membrane 9), and edema **(E)** around the capillary space with destructive red blood cell (RBC) in the lumen (original magnification × 6,000). **(C)** The adipocyte with signs of fat fragmentation, smaller drops of fat in the cytoplasm of adipocytes, lipid-laden phagolysosomes (LPN) and detective ring-like mitochondria (original magnification × 6,000). **(D)** Well preserved capillary endothelial cells with destructive erythrocyte (RBC) adhered to endotheliocytes in the lumen surrounded with few adipocytes without signs of fat fragmentation (FF) (original magnification × 6,000). Red dots mark ring-like mitochondria.

To assess the effect of H_2_S on the adaptive changes of mesenteric white adipocytes in aged rats fed with HFD, the treatment by NaHS and exposition to WIRS was used. Representative images of various kinds of changes in white adipocytes in aged rats fed with HFD with belonged stromal-vascular cells are shown in Figure 3. The differences of mesenteric white adipocytes in aged rats fed HFD and vehicle were observed (Figure 3A, B) in comparison to the group of aged rats fed HFD with NaHS treatment (Figure 3C, D). The adipocyte in aged rats fed HFD and vehicle were with signs of fat fragmentation and disrupted basal membrane, with many small lipid drops in the cytoplasm and different shaped mitochondria (Figure 3A). Many smallest peripheral lipid droplets are present in the marginal cytoplasm of adipocytes with defective mitochondria and lipid-laden phagolysosomes. Adipocyte collagen fibers were disorganized, and the changes in the microarchitecture of the capillary endothelial cells which have microvilli were observed (Figure 3B). The destruction of the capillary basal membrane and submembrane edema with destructive red blood cells in the lumen was detected (Figure 3B). These results suggested that HFD affects MA, inducing its defragmentation, mitochondrial dysfunction, and endothelial damage in aged rats.

The results of exogenous stimulation of H_2_S by NaHS showed that the adipocyte had fewer signs of fat fragmentation (smaller drops of fat in the cytoplasm of adipocytes) and showed a tendency to decrease the number of its detective mitochondria (Figure 3D). Under the changes induced by NaHS, well-preserved capillary basal membrane, endothelial cells, and destructive erythrocyte in the lumen were detected in the microvessel (Figure 3E). Our data showed that HFD and age-related alterations of MA, its mitochondria, and mesenteric connective tissue are reversible under the influence of H_2_S donor—NaHS. There was a remarkable difference in mesenteric endotheliocytes’ condition that confirms the cytoprotective effect of H_2_S donors.

**FIGURE 4:**
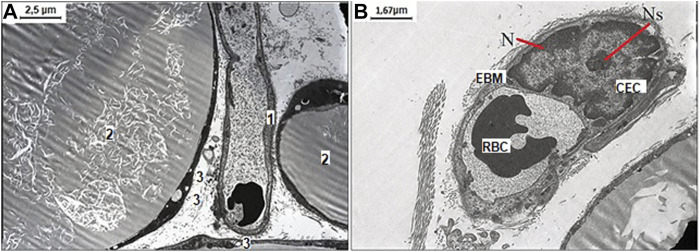
Representative pictures of the effect of NaHS fructose-fed and stress induction on ultrastructural examination of mesenteric material harvested from the area associated with the small intestine of aged rats. **(A)** The well-preserved capillary 1) between three white monovacuolar adipocytes 2) with single lipid droplets 3) (original magnification ×4,000). **(B)** The capillary with the endothelial cells (CEC) with an optimally developed nucleus (N) and nucleolus (Ns), and thickened and edematous basal membrane (EBM) with altered red blood cell (RBC) in its lumen (original magnification ×6,000).

After NaHS administration and WIRS induction, the enlarged monovacuolar fat cells were with a few smaller drops on the periphery. The released fat drops and well-preserved capillary without sign of endothelial dysfunction with an optimally developed nucleus and nucleolus are detected in interstitial space, which suggested mesenteric cells tended to recover under influence of increased H_2_S bioavailability (Figure 4).

#### NaHS, an H_2_S Releasing Donor, Reduces HFD Stimulated TBARS Production in Both Adult and Aged Rats Without and With Acute Stress

**FIGURE 5:**
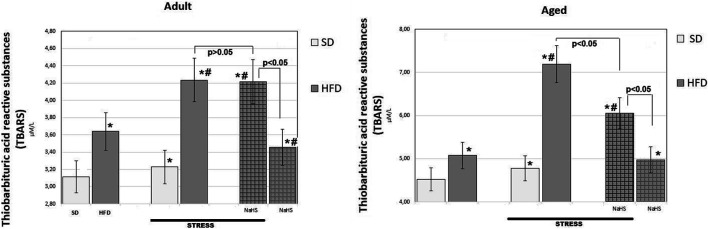
TBARS levels in adult and aged rats fed with a standard diet (SD) or high fructose diet (HFD) without and with H_2_S releasing therapy (NaHS) and induction of acute stress (*n* = 6); *p < 0.05 vs. SD; #*p* < 0.05 vs. HFD.

**FIGURE 6:**
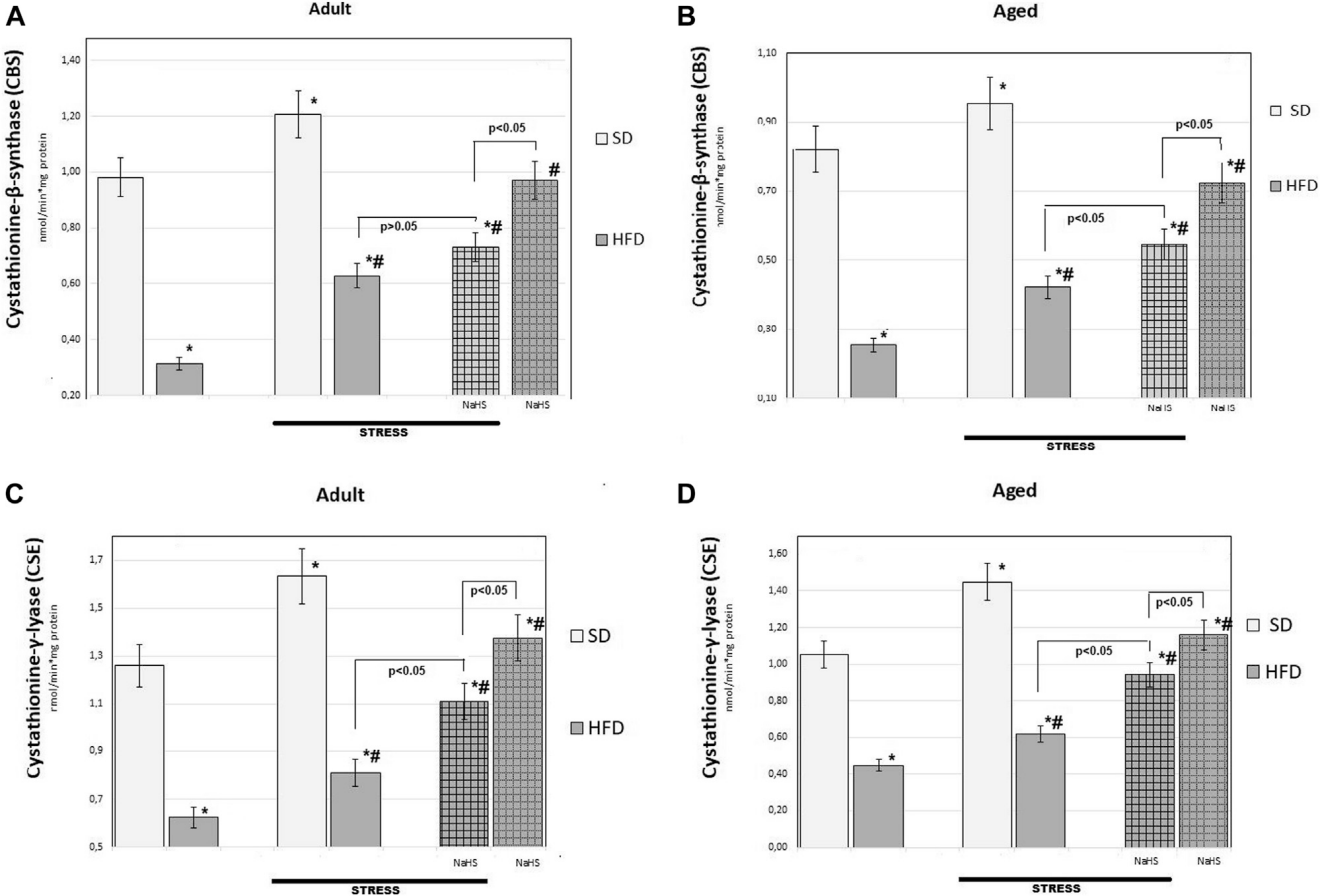
Activities of cystathionine-β-synthase (CBS) **(A,B)**, cystathionine-γ-lyase (CSE) **(C,D)** in adult **(A,C)** and aged rats **(B and D)** (*n* = 6) fed with a standard diet (SD) or high fructose diet (HFD) without and with H_2_S releasing molecule compound therapy (NaHS) and induction of acute stress; *p < 0.05 vs. SD; #*p* < 0.05 vs. HFD.

**FIGURE 7:**
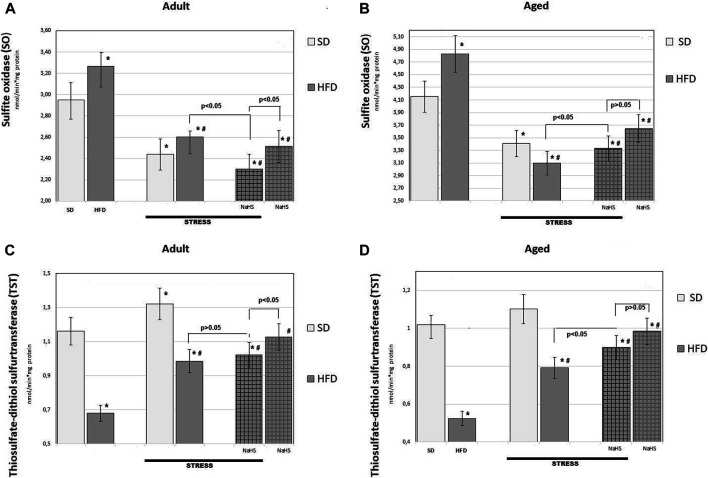
Activities of sulfide oxidase **(A,B)** and thiosulfate-dithiol sulfurtransferase (TST) **(C,D)** in adult **(A,C)** and aged rats **(B,D)** (*n* = 6) fed with a standard diet (SD) or high fructose diet (HFD) without and with H_2_S releasing molecule compound therapy (NaHS) and induction of acute stress; *p < 0.05 vs. SD; #*p* < 0.05 vs. HFD.

To further understand the exact stimulation effects of endogenous H_2_S on age- and HFD-related changes on oxidative damage in the mesentery, the TBARS levels (Figure 5) and CBS, CSE, SO, and TST activities (Figures 6, 7) involved in the biosynthesis of H_2_S mobilization were investigated.

In groups of adult and aged rats fed SD, the TBARS content was 3.11 ± 0.14 μM/L in adult rats and 4.52 ± 0.25 μM/L in aged rats (*p* < 0.01), we see an age-related increase in TBARS. In HFD-fed rats treated with saline, the levels of TBARS increased by 17% adult groups (3.64 ± 0.3 μM/L) and 12% aged groups (5.08 ± 0.17 μM/L) over results of the SD-fed groups (*p* < 0.05), and there was a significant increase in the level of TBARS in HFD. The effects of WIRS caused changes in the content of TBARS, and age differences were noted. Aged rats treated by being fed HFD under acute stress showed a 41% increase in TBARS (up to 7.19 ± 0.36 μM/L) and in adults, by 16% (up to 4.24 ± 0.15 μM/L) compared with animals without stress. In adult rats, administration of NaHS did not cause a significant reduction in TBARS in groups fed HFD without and with stress induction (*p* < 0.001). It was also found that conducting hydrogen sulfide modulation by the introduction of NaHS in aged rats, exposed to stress, significantly reduced TBARS by 16% compared with placebo-treated animals and without stress induction (*p* < 0.001). This effect of NaHS could be interpreted as anti-oxidative stress.

To further understand the exact influence of H_2_S donor administration, NaHS, on age- and HFD-related effects on mesentery, the expressions of CBS, CSE, TST, and SO activities involved in endogenous H_2_S mobilization were investigated (Figures 6, 7). In intact adult control rats on SD, the activities of CBS, CSE, TST, and SO were reaching 0.98 ± 0.06 nmol/min*1, 1.26 ± 0.08 nmol/min*1, 1.16 ± 0.09 nmol/min*1, and 2.9 ± 0.14 nmol/min*1 mg of protein, respectively. There were age-related differences in their enzyme activities. In intact aged rats on SD, the activities of CBS, CSE, TST, and SO were reaching 0.82 ± 0.06 nmol/min*1, 1.05 ± 0.07 nmol/min*1, 1.01 ± 0.04 nmol/min*1, and 4.15 ± 0.1 nmol/min*1 mg of protein, respectively. In HFD-fed groups, there were decreased enzyme activities (CBS, CSE, TST) and increased activity of SO in rats compared to SD-fed rats. There was also a significant difference in enzyme activities between adult and aged rats on HFD versus SD. Adult rats on HFD had much lower activity of CBS–69%, CSE–51%, and TST–53%; aged rats on HFD had much lower activity of CBS–70%, CSE–57%, and TST–50%, compared to the SD group (*p* < 0.05). The results showed increased activity SO in both groups: in adult rats up to 11% and in aged rats up to 15%. In contrast, the expression of CBS, CSE, and TST showed a tendency to decrease in the aged rats on HDF which have lower enzyme activities of CBS, CSE, and TST versus aged rats on SD. Notably, the increased activities of all H_2_S-related enzymes during induction WIRS were recorded, except for SO, which decreased (Figure 7A, B). We found that treatment by NaHS in adult rats exposed to stress on HFD resulted in increased activities of CBS–16%, CSE–37%, and TST by 5% over adult rats which did not receive NaHS (*p* < 0.01) and aged rats, CBS–28%, CSE–51%, and TST–13% (*p* < 0.01). These results indicate NaHS has the potential for regulating redox imbalance on mesenteric injury in rats induced by advanced age and HFD.

## Discussion

In modern times, the prevalence of metabolic disorders in the world has pandemic levels. Among them, obesity has similarly faced an upward trend, with the older population showing more susceptibility to obesity and related disorders than younger adults (Ponti et al., 2020). One interpretation of the ability of adipose tissue to reprogram whole-body physiology is their mitochondria which integrate several processes, including oxidative phosphorylation, ATP synthesis, and ROS generation that could cause metabolic signals for obesity Boudina and Graham (2014) and vascular disorders (Zhou et al., 2021). We have limited understanding of H_2_S signaling effects on metabolic disorders based on mesenteric adipocytes tissue damage and MA mitochondrial function, as well as other mesenteric vessels and fibroblasts. The physiological implication of H_2_S impacts our understanding of redox balance, cell homeostasis, and death, synthesis of pro-inflammatory molecules into the cytoplasm, and has potential for target therapy (Cedikova et al., 2016). In light of recent advances, the ultrastructural mitochondrial changes could be investigated by methods of classical transmission electron microscopy or 3D reconstruction of serial block-face scanning (Vincent et al., 2016). The electron microscopy results in the current experimental study demonstrating that age-related changes of mesenteric white adipocytes and endothelial condition are characterized by hypertrophic changes of MA with signs of fat degradation, different shaped and defective mitochondria, endothelial dysfunction, and abnormal basal membrane integrity. Numerous experimental studies have shown that epigenetic factors contribute to the risk of numerous metabolic disorders related to obesity, type 2 diabetes, and non-alcoholic fatty liver (Pichette et al., 2016; Melino et al., 2019). Both human and animal studies have reflected the potentially negative effects of glycemic and resistant carbohydrates оn metabolic physiology (Law et al., 2017; Henry et al., 2021; Omar et al., 2021). Consumption of a high amount of dietary fructose or other types of sugars in sweetened foods and aging could cause adipocyte tissue damage which might be related to metabolic and obesity-related disorders (Crescenzo et al., 2014; Pinnick et al., 2019). Human studies reveal the tendency of mesenteric fat wrapping or visceral adiposities and may act as a “red flag” in patients much earlier than symptom-onset (Li et al., 2021).

In the present study, we used animal models for induction metabolic disorders based on 28 days of consumption of HFD and compared age-related changes of mesenteric cells to rats fed with SD. The choice of induction of acute WIRS was important to study adaptive changes of mesenteric cells during acute injury. This model of WIRS was originally designed to study gastroprotection Takagi and Okabe (1968) and it quickly became widely useful for better understanding cytoprotective effects. Our report of results of MA ultrastructural study obtained from rats fed with HFD has shown mesenteric white adipocyte damage which is characterized by the disrupted basal membrane of MA, intracellular fat fragmentation with many smallest lipid drops in the cytoplasm, and different shaped mitochondria. There were signs of mesenteric capillary endothelial cell injury and fibroblast damage in adult rats fed with HFD during stress induction. These results confirm that metabolic disorders may cause similar changes as during accelerated aging (Salvestrini et al., 2019). The ultrastructural changes of MA in aged rats exhibiting HFD reflected ring-like mitochondria and many small drops of fat in the cytoplasm of adipocytes. Wide range effects of H_2_S signaling on the resolution of inflammation, inhibition of leucocyte-endothelial adhesion, mitochondrial dynamics in cell homeostasis, and redox balance have been extensively studied over the last 2 decades and implemented in novel therapeutic strategies (Papapetropoulos et al., 2020). Furthermore, a recent study demonstrated that stimulation of endogenous H_2_S biosynthesis can preserve adipocyte physiology in humans (Comas et al., 2019).

In our study, the dysfunctional mesenteric cells in aged rats fed with HFD induce increased TBARS production and decreased activity of H_2_S signaling was recorded, while stimulation endogenous H_2_S by NaHS caused MA and capillary endothelial cells’ cytoprotection, decreased TBARS level, and increased catalytic activity of H_2_S enzymes that regulate redox system via several intercellular and intracellular pathways (Pavlovskiy et al., 2018; Mezouari et al., 2020). These results for the first time indicate that H_2_S signaling is an important mesenteric mitoprotective factor that facilitates the dysregulation redox balance operated by intracellular and extracellular activities of CBS, CSE, TST, and SO. It would be of interest to study the defects in MA mitochondrial functions via mitochondrial redox balance by free radicals, glutamate/glutamine, indicators of programmed death, or the amount of ATP determination in the future. There are several explanations of the link between white adipocytes, inflammation, and redox balance which is essential to maintain metabolic homeostasis and structural-functional integrity of adipocytes, capillary endothelial cells, and fibroblasts in the mesentery (Lefranc et al., 2018; Ma et al., 2018; Magnuson et al., 2020). It has been suggested that dysregulated adipocyte-to-macrophage mitochondria transfer axis leads to obesity (Miliotis et al., 2019; Brestoff et al., 2020). Taken into account that, in the healthy state, secretory factors from adipocytes are responsible for preserving metabolism homeostasis and integrity in adipose tissue, our results show that endogenous H_2_S signaling in mesenteric white adipocytes is involved in age-related physiological changes. As such, these findings have potential as a therapeutic tool. It was interesting to note that stimulation production of endogenous H_2_S by NaHS demonstrated decreased mesenteric adipose tissue damage, mitochondrial impairment, and redox imbalance in aged rats with SD and both adult and aged rats exhibiting HFD.

In conclusion, the identification of the stimulation of endogenous hydrogen sulfide synthesis which inhibits mesenteric white adipocyte tissue, vessels, and fibroblasts damage after the overload of fructose in aged rats will be suggesting a possible future therapeutic application. Abolishment of mitochondrial dysfunction in the mesenteric adipocytes and stromal-vascular subcellular adaptive changes and redox imbalance seems to play an important role in the H_2_S effect on age-related and high fructose-induced mesenteric injury.

## Data Availability

The data from this study are not publicly available but are available from the corresponding author on reasonable request.

## Abbreviations

CECs: capillary endothelial cells; CBS: cystathionine beta-synthase; CSE: cystathionine gamma-lyase; H_2_S: hydrogen sulfide; HFD: high fructose diet; MA: mesenteric white adipocyte; NaHS: sodium hydrosulfide; NSAIDs: nonsteroidal anti-inflammatory drugs; SD: standard diet; SO: sulfite oxidase; TST: thiosulfate-dithiol sulfurtransferase; TBARS: thiobarbituric acid reactive substances; WIRS: water-immersion restraint stress.
